# Supplementary material for: Characteristics of Antibiotic Resistance and Tolerance of Environmentally Endemic Pseudomonas aeruginosa
Source: Antibiotics (Basel). 2022 Aug 18;11(8):1120. doi: 10.3390/antibiotics11081120 (PMC9404893; doi:10.3390/antibiotics11081120)
Supplement: Supplementary file 1 [file antibiotics-11-01120-s001.zip › antibiotics-1818779-supplementary.pdf]

Supplementary data

Title: Characteristics of Antibiotic Resistance and Tolerance of Environmentally Endemic *Pseudomonas aeruginosa*

Authors

Seryoung Kim [1], Satomi Masai [1], Keiji Murakami [2], Momoyo Azuma [3], Keiko Kataoka [4], Mayu Sebe [2], Kazuya Shimizu [5], Tomoaki Itayama [6], Niwooti Whangchai [7], Kanda Whangchai [8], Ikko Ihara [9], Hideaki Maseda [1, 10, 11*]

Affiliations

[1] Biomedical Research Institute, National Institute of Advanced Industrial Science and Technology, 1-8-31 Midorigaoka, Osaka 563-8577, Japan

[2] Department of Clinical Nutrition Faculty of Health Science and Technology, Kawasaki University of Medical Welfare, 288 Matsushima, Okayama 701-0193, Japan

[3] Department of Infection Control and Prevention, Tokushima University Hospital, 2-50-1 Kuramoto, Tokushima 770-8503, Japan

[4] Department of Microbiology and Genetic Analysis, Institute of Biomedical Science, Tokushima University Graduate School, Tokushima 770-8503, Japan

[5] Faculty of Life Sciences, Toyo University, 1-1-1 Izumino, Itakura-machi, Ora-gun, Gunma 374-0193, Japan

[6] Graduate School of Engineering, Nagasaki University, 1-14 Bunkyo, Nagasaki 852-8131, Japan

[7] Faculty of Fisheries Technology and Aquatic Resources, Maejo University, Chiang Mai 50290, Thailand

[8] Center of Excellence in Bioresources for Agriculture, Industry and Medicine, Chiang Mai University, Chiang Mai 50200, Thailand

[9] Department of Agricultural Engineering and Socio-Economics, Kobe University, Kobe 657-8501, Japan

[10] Graduate School of Life and Environmental Sciences, University of Tsukuba, 1-1-1 Tennodai, Ibaraki 305-8577, Japan

[11]Department of Environmental Engineering and Green Technology, Malaysia-Japan International Institute of Technology, Universiti Teknologi Malaysia, Kuala Lumpur 54100, Malaysia

*Correspondence to: Hideaki Maseda

Tel: +81-72-751-9810, Fax: +81-72-751-9628, E-mail: maseda.h@aist.go.jp

Table S1. The source of *P. aeruginosa* strains isolated from clinical settings

Isolation source	Strains	Number of strains
Blood	CL07, CL21, CL30, CL36, CL39	5
Catheter	CL02, CL06	2
Nasal mucosa	CL17	1
Oral mucosa	CL13	1
Pharynx secretions	CL03, CL04, CL24, CL25	4
Pus	CL27	1
Skin	CL09	1
Sputum	CL01, CL08, CL10, CL11, CL12, CL14, CL15, CL18, CL20, CL22, CL23, CL26, CL28, CL29, CL32, CL35, CL38, CL41, CL42	19
Urine	CL05, CL16, CL19, CL31, CL33, CL34, CL37, CL40	8
Total		42

Table S2. The results of antibiotic susceptibility of the strains isolated from the environment.

Strain	MIC (μg/mL)							
	CHL	CIP	TET	IPM	ATM	AMK	RIF	MIN
EN-001	128	0.125	32	2	4	2	32	64
EN-002	1024	1	128	2	>32	2	32	256
EN-003	128	0.125	16	2	4	2	16	32
EN-004	256	0.25	64	2	8	4	32	64
EN-005	256	0.25	32	2	4	4	32	32
EN-006	256	0.25	32	1	4	4	32	32
EN-007	256	0.125	32	2	4	2	32	64
EN-008	256	0.25	32	2	8	4	32	64
EN-009	256	0.25	32	1	8	4	32	64
EN-010	256	0.25	32	2	4	4	16	32
EN-011	128	2	32	2	8	2	16	32
EN-012	256	0.125	32	1	4	2	32	32
EN-013	256	0.0625	32	1	4	2	32	32
EN-014	512	0.25	64	1	8	8	32	64
EN-015	256	0.125	32	1	4	2	16	32
EN-016	128	0.125	32	0.5	4	4	32	32
EN-017	128	0.25	32	2	2	2	16	32
EN-018	128	0.25	32	2	4	2	16	32
EN-019	128	0.25	32	2	4	4	32	32
EN-020	128	0.25	32	2	4	4	32	32
EN-021	128	0.25	32	2	4	2	16	32
EN-022	64	0.25	32	1	4	2	32	32
EN-023	128	0.125	16	1	4	2	16	32
EN-024	128	0.125	32	0.5	4	4	16	32
EN-025	128	0.125	16	1	4	2	16	32
EN-026	128	0.25	32	2	4	2	32	32
EN-027	128	0.25	32	2	8	4	32	32
EN-028	128	0.125	32	1	4	1	16	32

Strain	MIC (μg/mL)							
	CHL	CIP	TET	IPM	ATM	AMK	RIF	MIN
EN-029	128	0.25	32	1	8	4	16	32
EN-030	128	2	32	0.5	1	2	32	32
EN-031	128	0.125	32	1	4	4	16	16
EN-032	128	0.125	32	1	4	4	16	16
EN-033	128	0.125	16	1	4	2	16	16
EN-034	64	0.0625	16	1	2	4	8	8
EN-035	128	0.25	32	2	4	4	32	32
EN-036	128	0.0625	16	1	2	4	8	16
EN-037	128	0.125	16	1	4	4	32	16
EN-038	128	0.25	32	1	4	4	32	32
EN-039	128	0.125	32	0.5	2	4	16	32
EN-040	128	0.0625	16	1	2	4	8	16
EN-041	128	0.0625	16	1	2	2	8	16
EN-042	128	0.125	32	1	4	4	32	32
EN-043	128	0.125	16	0.5	2	4	8	16
EN-044	128	0.125	32	1	4	2	16	32
EN-045	128	0.0625	16	1	4	4	16	16
EN-046	128	0.0625	16	1	2	4	16	16
EN-047	128	0.125	32	1	4	4	32	32
EN-048	128	0.125	16	1	4	4	8	16
EN-049	128	0.125	32	1	4	2	32	32
EN-050	128	0.25	32	2	8	4	32	32
8380[a]	128	0.125	32	1	4	8	16	32
PAO1S[b]	64	0.125	>256	2	4	4	16	256

[a] The representative strain of *P. aeruginosa* isolated from the clinic.

[b] Standard strain of *P. aeruginosa,* which possess Tet^r gene for marker in genome.

*CHL, chloramphenicol; CIP, ciprofloxacin; TET, tetracycline; IPM, imipenem; ATM, aztreonam; AMK, amikacin; RIF, rifampicin; MIN, minocycline.

Table S3. The results of antibiotic susceptibility of the strains isolated from the clinical site.

Strain	MIC (μg/mL)							
	CHL	CIP	TET	IPM	ATM	AMK	RIF	MIN
CL01	>1024	4	64	32	16	2	32	128
CL02	128	>32	32	32	8	4	16	32
CL03	256	0.125	32	1	4	2	16	32
CL04	256	0.125	32	2	4	4	32	32
CL05	256	0.25	32	4	8	8	16	64
CL06	256	0.125	64	2	4	4	32	64
CL07	512	1	128	2	16	2	32	256
CL08	256	0.125	32	32	8	8	16	32
CL09	128	0.125	32	1	8	2	16	32
CL10	256	0.25	32	1	4	4	32	32
CL11	128	0.25	32	1	4	4	32	32
CL12	128	0.125	32	16	16	1	16	16
CL13	128	0.125	16	1	8	1	32	8
CL14	512	0.25	32	1	32	2	32	64
CL15	128	<0.0625	16	32	8	4	16	8
CL16	256	0.25	64	2	8	8	32	32
CL17	256	0.125	64	1	4	2	16	32
CL18	64	0.125	16	2	<0.25	8	16	8
CL19	256	0.25	32	1	4	4	32	32
CL20	256	0.25	32	1	8	4	32	32
CL21	256	0.25	64	2	8	8	32	64
CL22	128	0.125	32	2	4	2	32	16
CL23	64	0.25	32	4	8	2	16	32
CL24	256	0.125	32	1	8	4	16	32
CL25	256	0.25	64	2	16	4	16	32
CL26	512	32	64	1	16	8	16	64
CL27	256	2	128	32	>32	128	64	64
CL28	>1024	2	32	8	2	1	32	128

Strain	MIC (μg/mL)							
	CHL	CIP	TET	IPM	ATM	AMK	RIF	MIN
CL29	256	0.5	64	2	16	4	16	32
CL30	128	0.25	32	2	4	2	32	32
CL31	256	4	32	1	8	2	32	64
CL32	128	0.25	32	1	4	4	32	32
CL33	256	0.25	32	1	4	2	16	32
CL34	256	0.25	32	1	8	2	16	32
CL35	512	2	128	4	>32	16	32	>256
CL36	256	0.25	32	1	4	4	32	32
CL37	256	>32	64	2	4	16	16	64
CL38	<8	4	32	16	8	32	8	16
CL39	256	0.25	32	1	4	4	16	32
CL40	512	8	64	32	>32	8	16	128
CL41	<8	0.5	<0.5	4	4	32	16	<1
CL42	128	0.25	64	2	4	8	16	64
8380[a]	128	0.125	32	1	4	8	16	32
PAO1S[b]	64	0.125	>256	2	4	4	16	256

[a] The representative strain of *P. aeruginosa* isolated from the clinic.

[b] Standard strain of *P. aeruginosa,* which possess Tet^r gene for marker in genome.

*CHL, chloramphenicol; CIP, ciprofloxacin; TET, tetracycline; IPM, imipenem; ATM, aztreonam; AMK, amikacin; RIF, rifampicin; MIN, minocycline.

Table S4. The POT values, tolerance to carbapenem, and mutation frequency of environmental strains.

Strain	POT value		Tolerance (MBC^{AD}/MIC^{AD})	Mutation frequency
	POT1	POT2		
EN-001	88	0	>128	1.73E-08
EN-002	634	0	>128	2.17E-08
EN-003	136	0	>128	1.10E-08
EN-004	88	0	128	4.05E-08
EN-005	30	0	64	2.83E-08
EN-006	365	0	>128	1.35E-08
EN-007	88	0	128	1.64E-08
EN-008	94	0	64	1.47E-08
EN-009	94	0	64	1.25E-08
EN-010	886	4	>128	6.40E-08
EN-011	415	16	16	6.46E-06
EN-012	378	16	128	5.56E-08
EN-013	656	34	32	6.08E-08
EN-014	830	0	128	1.45E-08
EN-015	40	16	128	2.51E-08
EN-016	382	0	>128	4.98E-08
EN-017	886	4	128	2.88E-08
EN-018	886	4	>128	2.35E-07
EN-019	30	0	32	5.69E-08
EN-020	30	0	128	1.13E-08
EN-021	886	4	128	4.44E-08
EN-022	892	0	128	1.09E-08
EN-023	378	48	128	4.38E-08
EN-024	56	16	>128	2.51E-08
EN-025	362	48	>128	9.94E-08
EN-026	634	0	128	2.63E-08
EN-027	634	0	128	1.71E-08

Strain	POT value		Tolerance	Mutation frequency
	POT1	POT2	(MBC^{AD}/MIC^{AD})	
EN-028	830	16	>128	1.87E-08
EN-029	659	4	128	5.95E-09
EN-030	222	0	128	1.11E-08
EN-031	206	0	64	8.69E-09
EN-032	206	0	128	1.10E-08
EN-033	556	0	128	9.01E-09
EN-034	206	0	64	8.11E-08
EN-035	44	0	64	4.73E-08
EN-036	206	0	128	3.09E-08
EN-037	572	16	8	2.74E-08
EN-038	637	0	128	1.90E-08
EN-039	637	0	32	3.47E-08
EN-040	206	0	128	1.07E-08
EN-041	205	0	4	1.60E-08
EN-042	633	0	128	1.91E-08
EN-043	44	0	16	9.79E-09
EN-044	44	0	256	8.10E-08
EN-045	205	0	16	8.07E-09
EN-046	205	0	128	3.99E-09
EN-047	637	0	128	2.10E-08
EN-048	205	0	32	5.50E-09
EN-049	44	0	128	8.27E-09
EN-050	44	0	32	4.27E-08
8380[a]	310	16	-	-
PAO1S[b]	382	0	32	2.75E-08

[a] The representative strain of *P. aeruginosa* isolated from the clinic.

[b] Standard strain of *P. aeruginosa,* which possess Tet^r gene for marker in genome.

Table S5. The POT values, tolerance to carbapenem, and mutation frequency of clinical strains.

Strain	POT value		Tolerance	Mutation frequency
	POT1	POT2	(MBC^{AD}/MIC^{AD})	
CL01	974	18	-	3.49E-07
CL02	672	0	-	1.32E-08
CL03	656	50	4	1.53E-08
CL04	61	48	64	5.20E-08
CL05	636	0	64	3.13E-08
CL06	383	16	64	6.33E-09
CL07	10	0	4	1.96E-08
CL08	823	16	-	1.08E-08
CL09	643	0	128	3.04E-08
CL10	44	0	64	3.49E-08
CL11	706	0	128	2.64E-08
CL12	383	36	-	1.68E-08
CL13	706	16	128	1.57E-08
CL14	604	50	64	1.18E-08
CL15	634	0	-	6.92E-09
CL16	634	0	128	9.46E-09
CL17	367	0	>128	9.84E-08
CL18	887	16	16	1.42E-08
CL19	122	0	64	4.36E-08
CL20	71	0	128	2.15E-08
CL21	823	0	32	8.37E-09
CL22	623	0	128	1.60E-08
CL23	974	16	8	1.83E-08
CL24	392	48	32	2.01E-08
CL25	392	16	16	1.49E-08
CL26	199	16	64	1.59E-08
CL27	287	2	-	3.77E-08

Strain	POT value		Tolerance	Mutation
	POT1	POT2	(MBC^{AD}/MIC^{AD})	frequency
CL28	382	48	-	1.62E-08
CL29	392	16	64	1.52E-07
CL30	622	22	128	1.42E-08
CL31	636	52	64	1.62E-08
CL32	554	0	128	3.03E-08
CL33	360	0	64	6.19E-09
CL34	360	0	64	1.77E-08
CL35	823	0	64	1.79E-08
CL36	62	0	64	3.38E-08
CL37	307	0	4	5.14E-08
CL38	575	0	-	3.83E-06
CL39	108	0	>128	2.52E-08
CL40	383	36	-	5.45E-09
CL41	282	0	2	8.86E-06
CL42	125	0	32	1.72E-08
8380[a]	310	16	-	-
PAO1S[b]	382	0	32	2.75E-08

[a] The representative strain of *P. aeruginosa* isolated from the clinic.

[b] Standard strain of *P. aeruginosa,* which possess Tet^r gene for marker in genome.

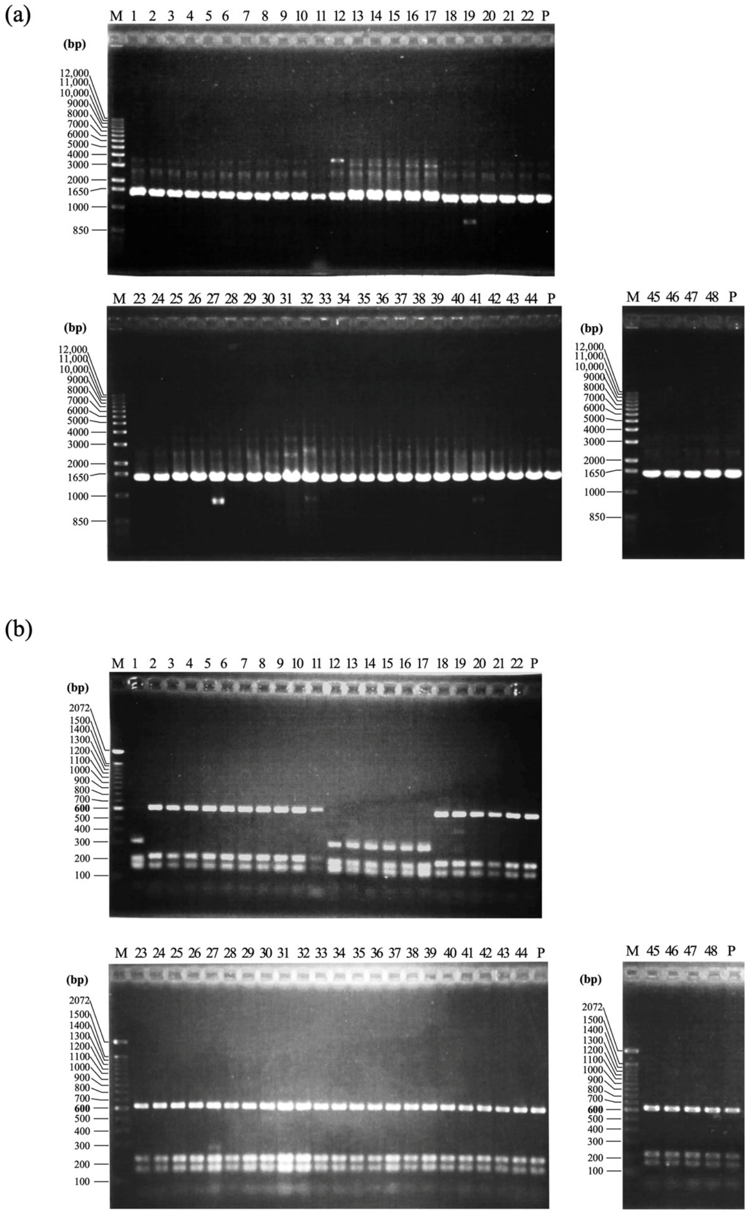

Figure S1. Electrophoresis diagram of DNA fragment amplified and restriction fragment length polymorphism analysis of 16S rDNA. (a) PCR analysis employing the primer BSF 8/20 and BSR1541/20 pairs on 16S rDNA. Lane M: 1 kb ladder; lane P: *P. aeruginosa* PAO1S; lanes 1 to 48: strains #1 to #48. (b) Restriction fragment length polymorphism analysis. Lane M: 100 bp ladder; lane P: *P. aeruginosa* PAO1S; lanes 1 to 48: strains #1 to #48.

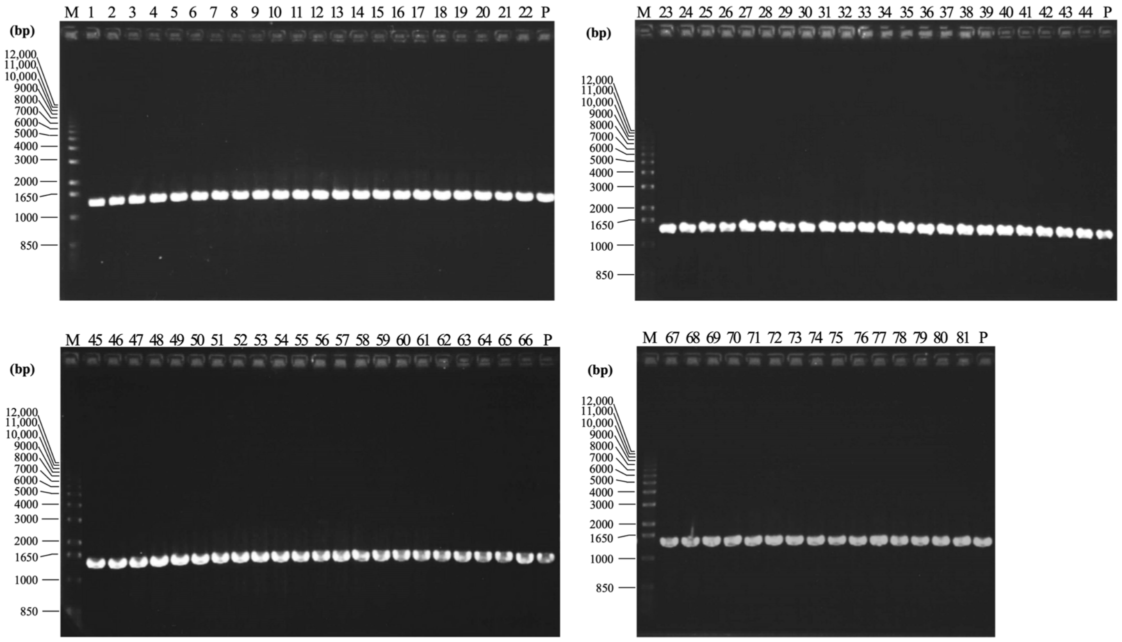

Figure S2. Electrophoresis diagram of DNA fragment amplified with primer set, 16S PAF 139/21 and BSR 1541/20, with genomic DNA of isolated bacteria from the environment. Lane M: 1kb ladder; lane P: *P. aeruginosa* PAO1S; lanes 1 to 81: strains isolated from river water, #1 to #81.

(a)

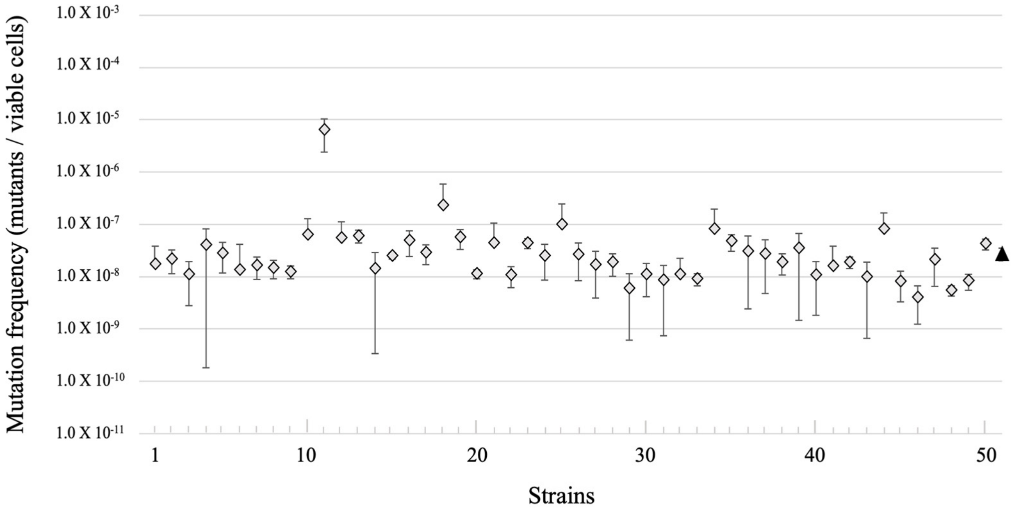

(b)

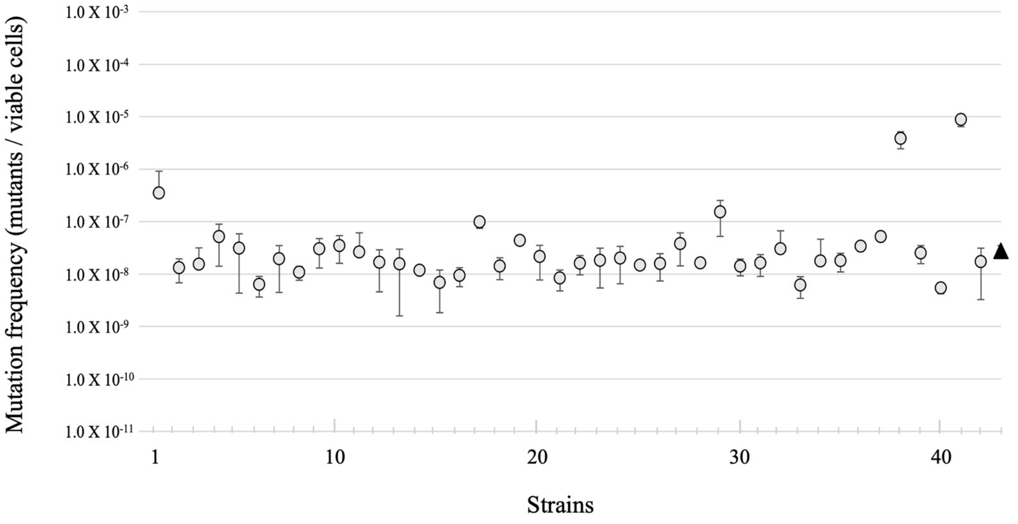

Figure S3. The mutation frequency ratio following exposure to rifampicin. (a) ◇ : the strains isolated from the environment, EN-001-050, Table S4. (b) ○ : the strains isolated from clinical settings, CL01-42, Table S5. ▲: PAO1S, which is the standard strain of *P. aeruginosa.*
